# Gaps between paediatric and psychiatric surveillance systems: rates of reporting in joint studies

**DOI:** 10.1192/bjb.2025.10122

**Published:** 2026-04

**Authors:** Andrew McWilliams, Hani F. Ayyash, Dasha Nicholls, Aditya Sharma, Michael Morton

**Affiliations:** 1 Department of Psychological Medicine, Institute of Psychiatry, Psychology & Neuroscience, King’s College London, London, UK; 2 Child and Adolescent Mental Health Services, Royal Free London NHS Foundation Trust, London, UK; 3 Specialist Children’s Health Services, Essex Partnership University NHS Foundation Trust, Southend-on-Sea, UK; 4 British Paediatric Surveillance Unit, Royal College of Paediatrics and Child Health, London, UK; 5 Division of Psychiatry, Imperial College London, London, UK; 6 Translational and Clinical Research Institute, Newcastle University, Newcastle upon Tyne, UK; 7 Cumbria, Northumberland, Tyne and Wear NHS Foundation Trust, Newcastle upon Tyne, UK; 8 Department of Health and Wellbeing, University of Glasgow, Glasgow, UK

**Keywords:** Epidemiology, child and adolescent psychiatry, liaison psychiatry, comorbidity, service development

## Abstract

**Aims and method:**

The British Paediatric Surveillance Unit of the UK Royal College of Paediatrics and Child Health contacts participating consultant paediatricians each month to survey whether particular rare conditions or events have been seen in their services. This national surveillance of rare paediatric events has allowed a large amount of research into multiple paediatric conditions. In 2009, the Royal College of Psychiatrists established a similar system – the Child and Adolescent Psychiatry Surveillance System (CAPSS) – to survey consultant psychiatrists in UK and Ireland. Since many conditions involve mental and physical health features, seven studies have been run using reporting to both systems, with simultaneous surveillance across both paediatricians and psychiatrists. Given the desire by policymakers, commissioners and clinicians for well-integrated physical and mental healthcare (‘joined-up working’), and if the surveillance systems were functioning well, the CAPSS Executive expected high rates of parallel reporting of individual patients to the two systems. The current study synthesises the rates of parallel reporting of cases to those two systems. We assimilate rates of parallel reporting across the seven studies using figures that have already been published, and by contacting contributing research groups directly where the relevant figures are not currently published. No new primary data were collected.

**Results:**

Of the 1211 confirmed cases, 47 (3.9%) were reported by both psychiatrists and paediatricians. No parallel reporting occurred in four of the seven studies.

**Clinical implications:**

Our findings raise questions about whether joined-up working in mental and physical healthcare is happening in practice. Research into challenges to obtaining comprehensive surveillance will help epidemiologists improve their use of surveillance and control for biases.

Surveillance methodologies allow researchers to study presentations of rare diseases and other uncommon healthcare events. As well as study of the diseases themselves, surveillance provides an opportunity to chart patient trajectories through healthcare systems, suggesting ways to improve screening, assessment processes and treatment, and also service development and policy more broadly.^1^ Near-comprehensive reporting coverage is necessary to ensure results are meaningful.

One such surveillance system is the British Paediatric Surveillance Unit (BPSU), established by the UK Royal College of Paediatrics and Child Health (RCPCH) to survey consultant paediatricians in the UK and Ireland.^2^ Research groups apply to the BPSU to survey for a particular event that they are interested in – such as a new presentation of a particular disease. The BPSU reviews the proposal and may ask for changes to be made. Once accepted, the BPSU runs the surveillance on behalf of the researchers, contacting consultant paediatricians each month over a set time period (often 12–18 months), to ask whether they have encountered the event. Initially this was achieved by sending cards out in the post, which were filled in and sent back by the paediatrician, but the system has now become fully electronic, with requests sent out by email and responses gathered online. Following this initial reporting, any consultant who notifies a case receives more detailed requests directly from the research groups for further information about the events being studied.

In 2009, the BPSU assisted the Faculty of Child and Adolescent Psychiatry of the UK Royal College of Psychiatrists (RCPsych) to establish a similar surveillance system for surveying consultant child and adolescent psychiatrists: the Child and Adolescent Psychiatry Surveillance System (CAPSS). It employs the same methodology as the BPSU, with researchers applying to run a study and the executive reviewing proposals. If approved, CAPSS then sends out monthly requests for notification to consultant child and adolescent psychiatrists. A number of important studies have now been run in CAPSS, details of which are contained in reports of the CAPSS Executive.^3^

Since many rare conditions present with symptoms and signs across both physical and mental health domains, it was recognised that studies might benefit from being run through the BPSU and CAPSS in parallel. Overall case ascertainment in surveillance is likely to be increased if multiple groups are encouraged to report,^4^ and parallel surveillance of paediatricians and psychiatrists expands the range of data that research groups can collect, capturing more aspects of physical and mental healthcare.

Seven studies have therefore been run using parallel BPSU and CAPSS surveillance (Box 1). For these studies, clinicians were given explicit instructions to report in parallel with their paediatric or psychiatric colleagues, each reporting to their respective surveillance system. With increasing attention from policymakers, healthcare providers and the media to promote integration of physical and mental healthcare (so-called joined-up working),^5,6^ joint surveillance studies might be expected to identify a number of individual patients simultaneously in both the paediatric and the psychiatric surveillance systems.

Box 1 Summary of aims of parallel paediatric and psychiatric surveillance studies

The British Paediatric Surveillance Unit (BPSU) and Child and Adolescent Psychiatry Surveillance System (CAPSS) have run seven simultaneous surveillance studies.

**Conversion disorder**

Aims: to estimate incidence of non-transient conversion disorder before 16 years of age, describe features of the presentation, current management and short-term outcomes, and inform service development.^25^

**Early-onset eating disorders**

Aims: to estimate incidence of eating disorders presenting before 13 years of age, describe features of the presentation, current management and short-term outcomes, and inform service development.^14,26–30^

**Children and adolescents with attention-deficit hyperactivity disorder in transition between children’s services and adult services (CATCh-uS)**

Aims: to describe features of transition between children’s services and adult services for children and adolescents with attention-deficit hyperactivity disorder, chart current patient trajectories management and short-term outcomes, and inform service development.^31,32^

**Childhood disintegrative disorder**

Aims: to estimate the incidence of childhood disintegrative disorder (CDD), describe features of the presentation, current assessment and management and short-term outcomes. This would enable more accurate prognostic information to be shared with families, allow more tailored service provision for affected children, assist in planning of services at a regional and national level, raise awareness of CDD among clinicians and enable planning of future studies of aetiology and interventions.^33^

**Gender dysphoria**

Aims: to estimate the incidence of gender dysphoria, describe features of the presentation, current support and short-term outcomes, and inform service development.^10,34^

**Sydenham’s chorea**

Aims: to estimate incidence of Sydenham’s chorea, describe features of the presentation, current management and 2-year outcomes, including complications and educational outcomes, raise awareness of the course of the disease and inform service development.^13,35^

**Avoidant/restrictive food intake disorder**

Aims: to estimate incidence of avoidant/restrictive food intake disorder (ARFID), compare rates, presentation and management with other countries, generate new priority research questions to inform decision-making and help match patient need with sufficient funding allocations.^36^

## Aims of current study

In this article, we synthesise rates of parallel reporting across the seven parallel surveillance studies. We then consider the reasons why these rates of parallel reporting might have occurred, discuss the implications for researchers planning surveillance with clinicians and consider what our findings suggest about the provision joined-up physical and mental healthcare in the UK and Ireland.

Principles of surveillance methodology have had only limited discussion in the child and adolescent psychiatry literature, so we believe our paper begins to plug that gap.

## Method

No new patient-level data were collected for the current report. Instead, we aimed simply to synthesise information about rates of parallel reporting, as provided by the research teams who had carried out parallel surveillance studies with the BPSU and CAPSS. We therefore gathered three figures for each of the jointly run studies: confirmed cases reported to the BPSU, confirmed cases reported to the CAPSS and confirmed cases reported to both systems. Where these figures were not already available in published reports, we contacted the research teams directly to request the information. Details of the seven studies are published on the BPSU website (www.rcpch.ac.uk/work-we-do/bpsu/past-studies).

Patients and the public were not involved in the design, conduct, reporting or dissemination plans of our research.

## Results

Results are shown in Table 1. Parallel reporting occurred in on 3.9% of cases (47/ 1211) across the 7 studies. No parallel reporting occurred at all in 4/7 studies. The study with the highest rate of parallel reporting was that of early-onset eating disorders, with parallel reporting happening for 34/208 cases (16.3%).

**Table 1:** Confirmed cases reported in simultaneous surveillance studies

Reported cases	Conversion disorder^25^	EOED^14^	CATCh-uS^31^	CDD^a^	Gender dysphoria^34,b^	Sydenham’s chorea^13^	ARFID^36,c^	Total
Total, *n*	204	208	315	34	80	43	327	1211
BPSU alone, *n* (%)	117 (57.3)	39 (18.8)	202 (64.1%)	19 (55.9)	11 (13.8)	43 (100)	191 (58.4)	622 (51.3)
CAPSS alone, *n* (%)	78 (38.2)	135 (64.9)	113 (35.9)	15 (44.1)	69 (86.3)	0	132 (40.4)	542 (44.7)
Both systems, *n* (%)	9 (4.4)	34 (16.3)	0	0	0	0	4 (1.2)	47 (3.9)

EOED, early-onset eating disorders; CATCh-uS, children and adolescents with attention-deficit hyperactivity in transition between children’s services and adult services; CDD, childhood disintegrative disorder; ARFID, avoidant/restrictive food intake disorder; BPSU, British Paediatric Surveillance Unit; CAPSS, Child and Adolescent Psychiatry Surveillance System.

a. Preliminary data at 12 months, personal communication, by email with Dr Michael Absoud, London, 2017.

b. Preliminary data at 9 months.

c. Personal communication, by email with Dr Javier Sanchez Cerezo, London, 2024.^36^

## Discussion

The seven presentations studied by joint surveillance (conversion disorder, eating disorders, attention-deficit hyperactivity disorder (ADHD), childhood disintegrative disorder, gender dysphoria, Sydenham’s chorea and avoidant/restrictive food intake disorder) involve frequent mental and physical signs and symptoms. Given the wish for joined-up mental and physical healthcare service provision, we expected a high proportion of cases to be reported by both consultant psychiatrists and paediatricians across the seven studies. However, dual reporting was rare overall – occurring on just 3.9% of occasions – and completely absent in four of the seven studies.

Globally, we know of only a few healthcare research studies that have attempted parallel surveillance via more than one reporting system. COVID-19 has been studied by the CoroNerve Studies Group, using reporting of adult cases from professionals working in neurology, stroke, psychiatry and intensive care.^7^ This was achieved through the major UK neuroscience bodies representing professionals, rather than by using a dedicated surveillance system. Multi-specialty reporting was carried out by the Australian Paediatric Surveillance Unit for conversion disorder and for eating disorders.^8,9^ This involved a multiprofessional group reporting to a single surveillance system. Even though conversion and eating disorders both involve significant mental health need, fewer than 4% of reporters there were psychiatrists. The reason for the low number of psychiatrists is unclear, but this illustrates how difficult it can be to make sense of data-sets without understanding the drivers behind reporting.

Our finding of low rates of parallel reporting could be due to a number of reasons. Clinicians might have chosen not to report in parallel because they believed this was the correct thing to do, despite instructions to report in parallel. This could happen if a disease is thought to fit better within one specialty than the other – creating a so-called ‘negative dependency’ in reporting. Alternatively, if patients are seen only by paediatricians and psychiatrists who are not consultants, or if local commissioning of healthcare means that psychologists, nurses or other professionals see patients, then cases will not be reported unless a consultant is aware of them. The locally variable age of transition to adult services will also affect parallel reporting. In one locality, a 17-year-old presenting to community mental health services may be seen by a child and adolescent psychiatrist, unless they have psychosis, in which case they might be under the care of a general adult psychiatrist in a first episode psychosis team. Similarly, if transition to adult physical care services occurs at age 16, then during an admission for lung disease the individual would not meet any paediatricians. Furthermore, psychiatric liaison during that admission might be provided by either child/adolescent or by adult psychiatrists, depending on local service commissioning. Surveillance methodologies need to account for this potential for geographical variability.

The reporting patterns might have been affected by differences between the two surveillance systems themselves. Return rates in one parallel surveillance study were 93% for the BPSU but only 76% for the CAPSS.^10^ In 2009, the BPSU had around 94% of consultant paediatricians signed up;^11^ this high coverage is due partly to the sharing of contact details of consultants within college databases. However, since there does not seem to be a more recently published figure it could be useful to update this. The exact coverage for the CAPSS is harder to establish. RCPsych census information from 2021 tells us there were 958 child and adolescent psychiatrists in the UK, including full-time, part-time and locums at that date.^12^ Set up from the start with opt-in participation, 695 consultants were on the CAPSS list in 2021, putting UK coverage at 72.5%.

In six of the seven studies, paediatricians and psychiatrists answered the same screening questions to determine whether they had seen a case (the exception being Sydenham’s chorea, where the CAPSS questions also asked about diagnoses that were not recent^13^). There may be reasons why paediatricians and psychiatrists are more or less likely to respond, as well as how they do this. Although both surveillance systems were initially set up with postal reporting,^14^ they have changed at different times to electronic reporting, via hybrid transitional periods. The BPSU was fully electronic by 2015, launching an updated web platform in 2022. The CAPSS was fully electronic by 2018. Electronic reporting may increase engagement for some, but decrease it for others who experience high email demand and email fatigue,^15^ which might tend to differ between psychiatrists and paediatricians. Our anecdotal experience suggests that paediatric trainees view participation in the BPSU as a ‘badge of honour’ that they will be awarded when they reach consultant-hood. Psychiatric trainees seem less aware of the CAPSS and the importance of participation.

### Considerations for clinicians and commissioners

Our finding should raise concern about whether children and adolescents with both physical and mental health needs are in fact being seen by both paediatricians and psychiatrists. In recent decades, increasing attention from policymakers, healthcare providers and the media has been directed at integration of physical and mental healthcare in general,^5,6^ including specifically for eating disorders^16^ and conversion disorder.^17^ Moves towards more joined-up working have been welcomed by patients, carers and clinicians.^18,19^ England’s new Rare Diseases Action Plan (2022) highlights particular challenges to addressing mental health need in rare conditions,^20^ and COVID-19 has had a significant mental health impact.^21,22^

Despite this, joined-up working does not always happen in practice, with mental and physical healthcare sometimes commissioned from teams working in different buildings, under different organisations and without shared healthcare records.^19,23^ Better evidence is required to guide service development, practice and evaluation^19^ and joint CAPSS/BPSU surveillance projects could provide valuable data. Promoting participation in CAPSS among psychiatrists at all stages of their training can only help improve the data.

### Considerations for researchers

Researchers will be interested in the factors that make the quality of surveillance data vulnerable. These factors should then be investigated directly, so that they can subsequently be controlled when planning future studies. Secondary analysis of previously collected responses could show how often two or more paediatricians working in different subspecialties or centres report to the BPSU in duplicate, or how often two psychiatrists report to the CAPSS. Profession-specific factors affecting reporting should also be investigated directly. Attributes related to knowledge, skills, working practice or even personality types may affect both engagement and interpretation of questions when providing responses. Qualitative research – such as with surveys or focus groups – could offer a window on some of these profession-specific differences, as well as suggest ways to improve engagement.

Since surveillance involves a finite reporting period, with a start and an end date, the lag time between referral and assessment by clinical teams is a factor affecting whether the same child or adolescent is seen by both specialties within the reporting period. Local service configuration and clinical practices affect healthcare trajectories, so surveillance researchers will benefit from a nuanced understating of these.^24^ Biases introduced by differences in age of transition to adult services are especially damaging for researchers studying events with peak incidence in late adolescence, where data collection is therefore significantly affected by differences in reporting for over 16-year-olds.

In conclusion, the use of parallel surveillance through paediatric and psychiatric systems offers potential for study of complex diseases affecting both physical and mental health. Understanding patient trajectories through complex real-world healthcare systems can suggest ways for service development. The most comprehensive surveillance will therefore bring about the greatest benefit, so it is important that surveillance researchers appreciate the real-world factors driving reporting and anticipate how to mitigate problems. We urge continued audit of the provision of integrated mental–physical healthcare to ensure that services development prioritises joined-up working.

## Data Availability

Data availability is not applicable to this article as no new data were created in this study. Details of the sources of the analysed data are given in the article.

## About the authors

**Andrew McWilliams**, Honorary Clinical Lecturer, Department of Psychological Medicine, Institute of Psychiatry, Psychology & Neuroscience, King’s College London, London, UK, and Consultant Child and Adolescent Psychiatrist, Child and Adolescent Mental Health Services, Royal Free London NHS Foundation Trust, London, UK. **Hani F. Ayyash**, Consultant Neurodevelopmental Paediatrician, Specialist Children’s Health Services, Essex Partnership University NHS Foundation Trust, Southend-on-Sea, UK, and Scientific Committee Member, British Paediatric Surveillance Unit, Royal College of Paediatrics and Child Health, London, UK. **Dasha Nicholls**, Professor of Child and Adolescent Psychiatry, Division of Psychiatry, Imperial College London, London, UK. **Aditya Sharma**, Clinical Senior Lecturer in Child and Adolescent Psychiatry, Translational and Clinical Research Institute, Newcastle University, Newcastle upon Tyne, UK, and Honorary Consultant Child and Adolescent Psychiatrist, Cumbria, Northumberland, Tyne and Wear NHS Foundation Trust, Newcastle upon Tyne, UK. **Michael Morton**, Honorary Clinical Senior Lecturer and retired Consultant Child and Adolescent Psychiatrist, Department of Health and Wellbeing, University of Glasgow, Glasgow, UK.
